# Vancomycin therapeutic drug monitoring and population pharmacokinetic models in special patient subpopulations

**DOI:** 10.1002/prp2.420

**Published:** 2018-08-28

**Authors:** Joaquim F. Monteiro, Siomara R. Hahn, Jorge Gonçalves, Paula Fresco

**Affiliations:** ^1^ Faculdade de Medicina da Universidade do Porto (FMUP) Porto Portugal; ^2^ Instituto de Investigação e Formação Avançadas em Ciências e Tecnologias da Saúde (IINFACTS) Instituto Universitário de Ciências da Saúde (IUCS) Gandra Portugal; ^3^ Instituto de Ciências Biológicas Curso de Farmácia Universidade de Passo Fundo (UPF) Passo Fundo Brasil; ^4^ Laboratório de Farmacologia Departamento de Ciências do Medicamento Faculdade de Farmácia da Universidade do Porto (FFUP) Porto Portugal; ^5^ I3S Instituto de Investigação e Inovação em Saúde Universidade do Porto Porto Portugal

**Keywords:** pharmacokinetics, special subpopulations, therapeutic drug monitoring, vancomycin

## Abstract

Vancomycin is a fundamental antibiotic in the management of severe Gram‐positive infections. Inappropriate vancomycin dosing is associated with therapeutic failure, bacterial resistance and toxicity. Therapeutic drug monitoring (TDM) is acknowledged as an important part of the vancomycin therapy management, at least in specific patient subpopulations, but implementation in clinical practice has been difficult because there are no consensus and agglutinator documents. The aims of the present work are to present an overview of the current knowledge on vancomycin TDM and population pharmacokinetic (PPK) models relevant to specific patient subpopulations. Based on three published international guidelines (American, Japanese and Chinese) on vancomycin TDM and a bibliographic review on available PPK models for vancomycin in distinct subpopulations, an analysis of evidence was carried out and the current knowledge on this topic was summarized. The results of this work can be useful to redirect research efforts to address the detected knowledge gaps. Currently, TDM of vancomycin presents a moderate level of evidence and practical recommendations with great robustness in neonates, pediatric and patients with renal impairment. However, it is important to investigate in other subpopulations known to present altered vancomycin pharmacokinetics (eg neurosurgical, oncological and cystic fibrosis patients), where evidence is still unsufficient.

**Abbreviations**
ABW: actual body weight
AME: American
AUC: area under concentration versus time curve
AUTL: area under the trough level
CF: cystic fibrosis
CHN: Chinese
Ctrough: trough concentration
CI: continuous infusion
CLcr: creatinine clearance
Clvan: vancomycin clearance
CNS: central nervous system
CSF: cerebrospinal fluid
ECMO: extracorporal membrane oxygenation
GFR: glomerular filtration rate
GoR: grade of recommendation
ICU: intensive care unit
IVT: intraventricular
JPN: Japanese
LoE: level of evidence
MIC: minimum inhibitory concentration
MRSA: methicilin‐resistant Staphylococcus aureus
PPK: population pharmacokinetic
RCT: randomized controlled trial
SIRS: systemic inflammatory response syndrome
TDM: therapeutic drug monitoring

## 1 INTRODUCTION

Vancomycin is the drug of choice for infections caused by methicillin‐resistant *Staphylococcus aureus* (MRSA),^1^ the most prevalent multidrug resistant pathogen in the world. Therapeutic drug monitoring (TDM) is acknowledged as an important part of the management strategy when treating patients with this agent: safe and effective use of vancomycin requires compliance with recommendations concerning loading dose, TDM and dosage reduction in renal impairment and in other pathophysiological conditions. The emergence of vancomycin‐resistant enterococci, and more recently, vancomycin‐resistant *Staphylococcus aureus*, is directly related to vancomycin underdosing and is a problem of particular concern worldwide.^1^ These facts turn urgent the need to develop strategies to improve both vancomycin prescribing and monitoring.

The aims of the present work were as follows: to define the state of art of vancomycin TDM, based on the three most recent guidelines from USA, Japan, and China, to review TDM in specials populations and resume population pharmacokinetic (PPK) models of vancomycin.

## 2 METHODOLOGY

The methodology followed was similar to that used by Ye and coworkers.^1^ Three guidelines were selected as the basis of this work: the American from 2009 (AME),^2^ the Japanese from 2013 (JPN)^3^ and the Chinese from 2016 (CHN)^4^ guidelines on vancomycin TDM. These guidelines were analyzed, compared, and recomendations were evaluated. Level of evidence (LoE) and grade of recommendation (GoR) used in this work are shown in Table 1. The LoE and GoR used are in agreement with the GRADE system,^5^ also used in the CHN guideline.^4^ For comparison purposes, the graduation scale used in AME^2^ and JPN^3^ guidelines were transformed by two researchers, independently, and then discussed for consensus.

**Table 1:** Level of evidence and grade of recommendation (using GRADE approach)^5^

Level of evidence (LoE)	Grade of recommendation (GoR)
A (High quality)	1 (Strong recommendation) 2 (Weak recommendation)
B (Moderate quality)	
C (Low quality)	
D (Very low quality)	

**Figure 1:**
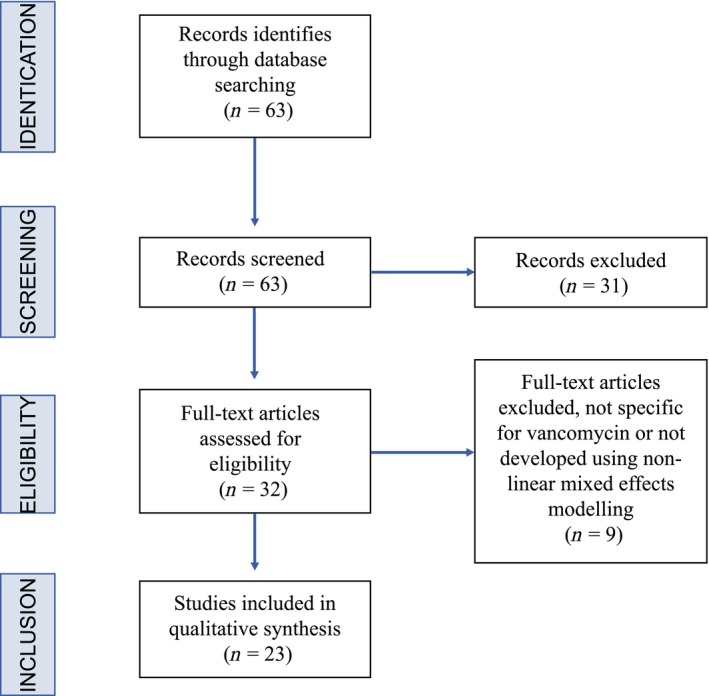
Population pharmacokinetic model review (PPK Model review) flow chart

The “Population Pharmacokinetic Model review” addressed the items “Dose adjustment method” and “Special populations” since these were the items less detailed in the referred guidelines. This review was carried out using Ovid MEDLINE and EMBASE electronic databases. The search equations used were: “Vancomycin” AND “Pharmacokinetic Model” for articles written in English, in humans until July 2017. A total of 63 records were found and two investigators independently screened the identified titles and abstracts to select articles. Of these, 31 records were excluded, either because they were not carried out in humans, do not directly concerned vancomycin or were general reviews of antibiotic use. The 32 full texted articles were tested for eligibility and nine were discarded since the models were not defined or were developed without using nonlinear mixed effects modelling. Finally, 23 articles were included in the qualitative synthesis (Figure 1), from now on referred as the “PPK Model review”.

## 3 THERAPEUTIC DRUG MONITORING

### 3.1 Indication and relevance

In the guidelines investigated, only two guidelines refers situations where TDM is recommended. All clinical conditions where TDM is indicated present low quality of evidence. Therefore, these are considered as strong recommendations.

The literature review gathered evidence for the recommendation of TDM in additional special population groups [hematologic, neurosurgery, extracorporal membrane oxygenation (ECMO), neonates and pediatric cystic fibrosis (CF)]. Table 2 summarizes data relative to the analysis of the clinical relevance of TDM for specific patient conditions.

**Table 2:** Guidelines review of TDM indication

Question	Answer (LoE/GoR)	Guideline (Reference)
Indication	1. TDM should be performed in patients who are likely to receive courses of more than three days. (B/1) 2. Intensive dosing, at high risk of nephrotoxicity, with serious infectious, unstable (deteriorating or improving) renal function, haemodialysis, obesity, low body weight and special conditions that cause fluctuating volumes of distribution. (C/1)	2013, JPN^3^
	1. TDM should be performed in patients who receive concomitant nephrotoxic agents, ICU admissions, obese patients and those who have burns or impaired renal function. (C/1) 2. TDM should be performed in elderly patients and patients with concomitant hepatic diseases. (C/2)	2016, CHN.^4^

CHN, Chinese; GoR, grade of recommendation; ICU, intensive care unit; JPN, Japanese; LoE, level of evidence; TDM, therapeutic drug monitoring.

Authors could not find additional data on the clinical relevance of TDM, other than that described by the CHN guideline.^4^ However, a meta‐analysis, including one randomized controlled trial (RCT) and five cohort studies, showed that TDM significantly increases the rate of clinical efficacy and decreases the rate of nephrotoxicity in patients treated with vancomycin.^6^ More recently, another RCT, comparing groups of patients treated with vancomycin (simple infections or infections with MRSA), with and without TDM also confirmed this conclusion.^7^ In fact, patients in the TDM group were discharged from the hospital more rapidly, reached clinical stability faster, had shorter courses of vancomycin and the time to initial target trough concentration was shorter. Moreover, in the MRSA infection subset, patients in the TDM group were also discharged from the hospital more rapidly, reached clinical stability faster, had shorter courses of vancomycin and attained initial target troughs in <5 days vs ≥5 days.^7^

Data on TDM implementation on hospitals is also scarce: in the last 10 years, only one paper concerning TDM implementation was identified. However, this paper describes that, in France, implementation of vancomycin TDM is quite high: vancomycin TDM was available in 97% (477/490) of hospitals.^8^ Unfortunately, a study carried out in Scandinavian countries showed that, in most cases, vancomycin TDM does not comply with recent recommendations/guidelines.^9^

Medical center implementation of the AME guideline,^2^ with associated training, resulted in a significant short‐term improvement in vancomycin dosing and TDM. The appropriateness of the prescribed dose increased from 51% of patients during the pre‐period to 78% during the postperiod (*P* < 0.0001). Similarly, overall appropriateness of sampling of vancomycin troughs at steady state improved from 36% to 55% (*P* < 0.03). Specifically, the appropriate timing of troughs (within 30 minute of the next dose) increased from 37% (64/173) during the pre‐period to 78% (149/191) during the postperiod (*P* < 0.0001).^10^

Another study claims that identification of improvement opportunities in TDM methology and implementation over a 1‐year period allowed a 37.5% reduction in inappropriately held vancomycin doses, although about 10% of doses remained as held inappropriately.^11^ Moreover, this study highlights the difficulties in identifying barriers to change and modify healthcare worker behaviour.^11^

### 3.2 Dose adjustement methods

Methods for dose adjustment discussed in the guidelines are summarized in Table 3.

**Table 3:** Guidelines review of dose adjustment methods

Question	Answer (LoE/GoR)	Guideline (Reference)
Dose adjustment methods	It should be noted that currently available nomograms were not developed to achieve targeted endpoints. Dose adjustments based in individual pharmacokinetic and verification of serum target achievement are recommended. (B/1)	2009, AME 2
	Vancomycin dosage should be administered and adjusted individually based on population pharmacokinetic methods (D/2)	2016, CHN.^4^

AME, American; CHN, Chinese; GoR, grade of recommendation; LoE, level of evidence.

Although the guidelines for TDM of vancomycin do not recommend the use of nomograms, a novel vancomycin dosing nomogram has been recently developed and validated at two Canadian teaching hospitals by Thalakada and co‐workers.^12^ This nomogram was considered a useful tool that clinicians can use in selecting appropriate initial vancomycin regimens based on age and serum creatinine, to achieve high‐target levels of 15‐20 mg/L. The authors, however, stressed out that this tool should not replace clinical judgment for patients with unstable and/or reduced renal function.^12^ Moreover, creatinine clearance‐based nomograms for individualizing vancomycin doses should be used with caution in patients who require substantially prolonged drug exposure such as those with infective endocarditis.^13^

The linear regression and Bayesian methods estimate, in general, more accurate dosage regimens. However, these methods require additional resources, such as information technology and healthcare personnel with background training in pharmacokinetics. The Bayesian methods offer additional advantages such as calculation of doses based on a single‐serum concentration and optimization of the patient's previous pharmacokinetic data to determine subsequent dosage regimens. Computerized programs, using the Bayesian estimation procedures, are able to achieve target concentrations in a greater percentage of patients, earlier in the course of therapy, than the empiric trough concentrations (Ctrough) and population methods.^14^, ^15^

The “PPK Model review”, in the adult population, found only six studies and, after analysis of the complete publicatons, three were excluded because they were not population models or were not developed using the nonlinear model of mixed effects methods. Table 4 describes the models found for the adult population in the mentioned review.

**Table 4:** Population pharmacokinetic models developed for adults

N	Pharmacokinetic Model	Pharmacokinetic Parameters	Covariates	Reference
72	One‐compartment	Cl = 4.90 L/h (if Clcr ≥ 80 mL/min); Vd=47.76 L	Clcr (if Clcr < 80 mL/min): Cl = 0.0654 × Clcr	16
106	Two‐compartment	Cl = 3.95 L/h (if Clcr ≥ 85 mL/min);	Clcr (if Clcr < 85 mL/min): Cl = 0.0339 × Clcr + 0.243	17
		Healthy volunteers V1 (L) = 0.205 × WT V2 (L = 43.4		
		Pneumonia V1 (L) = 0.720 × WT V2 (L) = 78.0		
		Bacteremia V1 (L) = 0.313 × WT		
		Other infections V1 (L) = 0.523 × WT V2 (L)=43.4		
398	Two‐compartment	Cl = 2.99 L/h V1 = 0.675 L/kg V2 = 0.732 L/kg	Cl = 2.99 + 0.0154 × Clcr ABW (covariate of V1 and V2)	18

ABW, actual body weight; Cl, vancomycin clearance; Clcr, creatinine clearance; N, sample size; V1, volume of the central compartment; V2, volume of peripheric compartment; Vd, volume of distribution; WT, weight.

### 3.3 Special populations

The need for clearer guidelines regarding vancomycin dosing and TDM for patient subpopulations has been recently reported.^19^

#### 3.3.1 Critically ill patients

The selected guidelines do not define special recommendations for this subpopulation. However, the literature review found 13 studies which are discussed below.

Significant challenges in vancomycin use in critically ill patients have been recently identified.^19^ There is wide variability in reported practices for antibiotic dosing and monitoring. Therefore, research to develop evidence‐based guidelines to standardize practices in critically ill patients is urgently needed.^20^

These patients may present very large volume distribution (Vd) as well as supranormal drug clearance.^21^, ^22^ Augmented renal clearance has frequently been observed in critically ill patients which was strongly associated with vancomycin pharmacokinetics. As a consequence, two‐thirds of these patients present subtherapeutic vancomycin concentrations.^23^

Long duration of fasting and massive diarrhea have been associated with elevations in serum vancomycin concentrations, which suggest that TDM might be necessary during enteral vancomycin administration in critically ill patients.^24^ Less than 40% of these patients attained therapeutic trough serum concentrations during the first 3 days of therapy.^19^ Patients with augmented renal clearance presented lower serum Ctroughs despite receiving higher maintenance doses and several loading doses.^19^ Subjects requiring intensive care unit support are significantly more likely to have higher vancomycin 24‐hour area under the concentration versus time curve (AUC24) and AUC than those who do not need intensive care unit support. Although vancomycin serum Ctroughs are predictive of vancomycin AUC, suboptimal exposure of vancomycin occurred in almost 20% of critically ill patients, despite Ctroughs being within the target range. To ensure optimal AUC/MIC, especially in critically ill patients, estimation of the AUC should be mandatory.^25^

Loading dose and target Ctrough for this population, achieved based on recommendations published in the literature, were lower than expected.^26^ Switching from intermittent to continuous infusion (CI) provided higher target attainment rates, a more robust drug exposure, a more rapid achievement of targeted drug levels with fewer subtherapeutic vancomycin levels observed,^27^, ^28^ cheaper and logistically more convenient, less TDM and less nephrotoxicity.^21^ Furthermore, CI yielded stronger concentration‐AUC correlations facilitating a single sample TDM strategy with AUC targets. A switch to CI may, therefore, improve clinical outcomes in vancomycin‐treated critically ill patients.^22^, ^29^

Although optimal administration based on PPK analysis and/or a Bayesian method has improved prediction accuracy, serum concentrations of vancomycin in patients with sepsis often deviate significantly from predicted values. Systemic inflammatory response syndrome (SIRS) duration was identified as influencing vancomycin concentration. Modifying dosing according to SIRS duration will improve prediction accuracy of vancomycin concentration based on TDM.^30^

Table 5 describes the two studies proposing PPK models for critically ill patients found in the “PPK Model review”.

**Table 5:** Population pharmacokinetic models developed for critically ill patients

N	Pharmacokinetic model	Pharmacokinetic parameters	Covariates	Reference
206	One compartment	Cl = 4.6L/h Vd = 1.5L/kg	Cl = 4.6 × Clcr/100 ABW (covariate of Vd)	31
46	One compartment	Cl = 0.86 mL/min/kg Vd = 1.69 L/kg	Cl = 0.872 − 0.015 × age (years) − 0.007 × ApII + 0.234 × Ab + 0.346 ClcrL (mL/min/kg) ABW (covariate of Vd)	32

Ab, serum albumin (g/dL); ABW, actual body weight; ApII, APACHE II score; Cl, vancomycin clearance; Clcr, creatinine clearance; ClcrL, creatinine clearance by the Levey formula; N, Sample size; Vd, volume of distribution.

#### 3.3.2 Pediatric patients

Only the JPN guideline^3^ presented evidence on the advantages of TDM on pediatric patients (Table 6).

**Table 6:** Guidelines review of pediatric patients' considerations

Question	Answer (LoE/GoR)	Guideline
Pediatric patients	1. First trough concentration can be obtained before the fourth dose (on day 2 if administered every 6 h). (C/1) 2. Vancomycin 15 mg/kg every 6 h is recommended for infants and children, and doses should be adjusted according to the result of TDM. Although few data are available to guide the dosing regimen in adolescent patients of ≥12 years old, doses of 15 mg/kg may be given every 8 h. (C/1) 3. To date, there are limited data to support the efficacy and safety of targeting trough concentrations of 15–20 mg/L in children, and additional study is required. (Unresolved issue)	2013, JPN^3^

GoR, grade of recommendation; JPN, Japanese; LoE, level of evidence; TDM, therapeutic drug monitoring.

Current recommended vancomycin dosing regimens in pediatric patients (40‐60 mg/kg/day), result frequently in subtherapeutic concentrations.^33^ Febrile neutropenia, a significant risk factor for augmented renal clearance in this subpopulation, indirectly influenced vancomycin clearance (Clvan) due to increased glomerular filtration rate (GFR). Increasing the initial dose is, therefore, required for achieving optimal therapeutic concentrations in pediatric patients with febrile neutropenia.^34^ The probability of achieving an AUC/MIC >400 using only one trough serum concentration and one minimum inhibitory concentration (MIC) in patients receiving 15 mg/kg every 6 hours is variable according to the method used to calculate AUC. In children, an AUC/MIC of 400 correlates with a Ctrough of 11 mg/L using a trapezoidal method to calculate AUC.^35^

For pediatric patients, monitoring of vancomycin Ctroughs is a recommendation stated in the summary of product characteristics and by several professional societies.^3^ During a study where vancomycin TDM was performed and 7935 vancomycin concentrations were obtained, the median Ctrough increased from 10.9 to 13.7  mg/L,^36^ which agrees with the recommendations published by the Infectious Disease Society of America.^2^ These data suggest that vancomycin TDM is commonly performed in pediatric patients, and the majority of abnormal Ctroughs are associated with appropriate modifications of the dosing regimen.^36^ Nevertheless, vancomycin TDM practices are reported to be highly variable in children admitted to pediatric hospitals.^37^ The frequency with which serum vancomycin concentrations were monitored in children increased after the publication of the adult guidelines. This fact made some authors claim that the development of pediatric consensus guidelines is needed to optimize patient care and resource utilization.^37^

Pediatric PPK models for vancomycin with Bayesian estimation can be used to reliably predict vancomycin exposure in children: the use AUC instead of Ctroughs, alone, can maximally optimize vancomycin administration in children.^41^ Compared with one sample, the two samples sampling strategy improved accuracy and precision in estimating and predicting future AUCs.^40^

Overweight and obese pediatric patients may have elevated initial vancomycin Ctroughs when empiric dosing is based on total body weight. This fact should make TDM mandatory in children.^38^

In pediatric cancer patients, a Vd of 34.7 L was reported and clearance values that were correlated with body weight, tumor disease, and cyclosporine co‐administration.^39^ Based on simulation results, dose (mg/kg) should be individualized based on body weight and cyclosporine co‐administration.^39^

The “PPK Model review” in pediatric and neonatal patients found five and seven studies, respectively. From these, three were selected in pediatric and six in neonates. The excluded studies were those that were not population models or were not developed using nonlinear modeling of mixed effects methods (Table 7).

**Table 7:** Population pharmacokinetic models developed for pediatric and neonate patients

Patients	N	PK model	PK parameters	Covariates	Reference
Pediatric	15	Two‐compartment	Cl V1 = 0.27 L/kg k_12_ = 1/h; k_21_ = 0.59/h	Cl = 0.018 × (ABW/70) + 0.460 × Clcr LBMcorrected (covariate of V1)	41
	6	Two‐compartment	Cl = 0.11 L/h/kg V_ss_ = 0.63 L/kg t_1/2_ alfa = 0.8 h; t_1/2_ beta = 5.63 h	ABW (covariate of Cl and Vss)	42
	78	Two‐compartment	Cl = 0.1 L/h/kg V1 = 0.27 L/kg V2 = 0.16 L/kg Cl_distribution_ = 0.16 L/h/kg	ABW	43
CF	67	One‐compartment	Cl = 5.57 L/h/70 kg; Vd = 44.1 L/70 kg	ABW	44
Neonates	152	One‐compartment	Cl = 0.068 L/h/kg Vd = 0.62 L/kg	ABW; Clcr; PMA	45
	249	One‐compartment	Cl = 0.276 L/h Vd = 1.75 L	Cl (L/h) = 0.345 (WT/2.9 kg)^0.75^ × F_mat_ × (1/Cr_mg/dl_)^0.267^ F_mat_ = 1/(1 + [PMA_wk_/TM_50_]^−Hill^) Vd (L) = 1.75 (WT/2.9 kg)	46
	70	One‐compartment	Cl = 0.066 L/h/kg Vd = 0.572 L/kg	– PMA and co‐administration of amoxicillin‐clavulanic acid (covariate of Cl) – Co‐administration of spironolactone (covariate of Vd)	47
	374	Two‐compartment	Cl = 0.066 L/kg Vdss = 0.79 L/kg	– WT, Cr (covariate of Cl) – Postnatal age and prematurity (<28 weeks) (covariate of Vd)	48
	47	One‐compartment	Cl = 0.276 L/h Vd = 1.75 L	Clcr and postnatal age (covariates of Cl)	49
	134	One‐compartment	Cl = 0.18 L/h; Vd = 1.7 L	Cl =0.18 × (WT/2.5)^0.75^ × (0.42/Crs)^0.7^ × (PMA/37)^1.4^ Vd = 1.7 × (WT/2.5)^1^	50

ABW, actual body weight; Cl, vancomycin clearance; Clcr, creatinine clearance; Cr, creatinine; Fmat, maturation function; Hill, coefficient of Hill; LBM, lean body mass; N, sample size; PMA, postmenstrual age; TM50, PMA when maturation reaches 50% adult clearance; V1, volume of central compartment; V2, volume of peripheric compartment; Vd, volume of distribution; Vss, volume of steady state; WT, weight.

- With cystic fibrosis
  No reference concerning pediatric patients with CF was found in the three international guidelines evaluated.
  The “PPK Model review” revealed only two studies concerning this population subgroup. One of these showed that vancomycin dosing of 60 mg/kg/day does not reliably achieve a vancomycin Ctrough of 15‐20 mg/L in pediatric patients with CF.^50^ The second also reported that younger CF patients may require higher vancomycin doses.^51^
  The PPK model found for this specific subpopulation is described in Table 7.
- Neonates
  In neonates, vancomycin is the first choice for late‐onset sepsis treatment. However, prescribing the right dose and dosing regimen remains a challenge in neonatal intensive care units.^52^ The high degree of pharmacokinetic variability in neonates makes TDM essential to ensure adequate therapeutic exposure^53^ and prevent adverse renal outcomes.^54^
  When using TDM in neonates the basic rules apply. However, additional factors should also be taken into consideration. First, due to both pharmacokinetic variability and nonpharmacokinetic factors, the correlation between doses and concentration is poor, but can be overcome using more complex, validated dosing regimens. Second, the time to reach steady‐state is increased, especially when no loading dose is used and, therefore, TDM sampling timing is of utmost importance in neonates. Third, the target concentration may be uncertain. Finally, because of differences in matrix composition (eg, protein, bilirubin), assay‐related inaccuracies may differ in neonates.^55^ With currently recommended vancomycin dosing, the therapeutic target of AUC/MIC > 400 is achieved only by 25% of neonates.^56^ Most of Ctrough in neonates achieved using two published dosing regimens did not reach the 10 mg/L.^57^ These results illustrate the urgent need for prospective validation of neonatal vancomycin dosing regimens.^57^
  Several vancomycin dosing schedules have been proposed, mainly based on neonate's age (both postmenstrual and postnatal), body weight or serum creatinine level. Other covariates [eg, ECMO, indomethacin/ibuprofen, and growth restriction] of vancomycin pharmacokinetics have been reported in neonates. Because age or weight is the most relevant covariates of renal maturation, these should be considered first in neonatal vancomycin dosing guidelines and further adjusted by renal dysfunction indicators (eg, ECMO and ibuprofen/indomethacin).^58^
  There is no consensus on vancomycin dosing in newborns and young infants. The empirical dosing method used was found inadequate in one‐third of patients.^59^ A simplified schedule of vancomycin seemed to lead to achieving target drug concentrations in most patients while avoiding renal toxicity.^60^ CI in neonates is well tolerated, require less blood sampling and may result in improved attainment of target concentrations.^61^ A patient‐tailored optimized dosing regimen should be routinely used to individualize vancomycin continuous administration.^62^ Several authors anticipate that complex validated dosing regimens, with subsequent TDM sampling and Bayesian forecasting, are the next step in individualizing therapy in neonates.^55^
  Modeling and simulation approaches have clear advantages in dosing optimization of antimicrobial agents in neonates.^63^ Pharmacometric modeling and simulation approaches allow to characterize population average, pharmacokinetic parameters, intra and intersubject variability, and to identify and quantify key factors that influence antibiotics pharmacokinetic behavior during the neonatal period.^64^ Simulations showed that the maintenance dose should be adjusted more precisely to each neonate based on weight and serum creatinine values.^65^ A model‐based vancomycin dosing calculator has been integrated in routine clinical care in several neonatal intensive care units. In this proof‐of‐concept study evidence for integrating model‐based antimicrobial therapy in neonatal routine care is provided.^66^ Monte Carlo simulations based on this PPK model suggest that vancomycin dosing guidelines based on serum creatinine concentration have a greater likelihood of achieving Ctroughs of 5‐15‐mg/L compared with other dosing regimens.^50^
  Table 7 describes the studies proposing PPK models for neonates' patients found in the “PPK Model review”.

#### 3.3.3 Elderly patients

The selected international guidelines also do not present specifications for this subpopulation. In the “PPK Model review” three studies were found concerning elderly patients.

The recommended target range of 15‐20 mg/L for vancomycin Ctrough seems to be acceptable for controlling vancomycin exposure, although a value of approximately 11 mg/L was found as optimal and safer in elderly patients.^67^ Efficacy of vancomycin was associated with area under the trough level (AUTL), a novel pharmacokinetic parameter.^68^ Determining the target AUTL or Ctrough may enhance the efficacy of vancomycin therapy in elderly patients with MRSA pneumonia.^68^ Given that nephrotoxicity may increase with a Ctrough >15 mg/L, this level should not be exceeded in this subpopulation.

#### 3.3.4 Obese patients

The guidelines point the use of actual body weight (ABW) for dose calculation but do not refer whether any adjustment is required for TDM in the obese patients' subpopulation. The “PPK Model review” allowed us to identify eight studies in this subpopulation.

Vancomycin dosing protocol led to the attainment of therapeutic Ctroughs in only 35.4% of obese patients.^69^ Moreover, overweight and obese pediatric patients may have elevated initial vancomycin Ctroughs when empiric dosing is based on ABW and, therefore, TDM should be mandatory for this specific subpopulation.^38^

Vancomycin TDM showed that underdosing and overdosing occur more often and effective levels are less often achieved, in obese patients. TDM might be of special importance, in obese patients.^70^ The majority of these patients present subtherapeutic concentrations, which increases the risk of treatment failure and bacterial resistance. Further studies are needed to determine the optimal dosing strategy in morbidly obese patients, ie with more than 100 kg and at least 140% of their ideal body weight.^69^ Calculating individual pharmacokinetic parameters using equations may be a valid tool for dosing vancomycin in obese patients with renal insufficiency.^71^

TDM has been correlated with pharmacokinetic/pharmacodynamic optimization for vancomycin in the obese population with skin and soft tissue infections, and should be used in these cases.^72^ Using two serum vancomycin concentrations significantly improves subsequent target Ctrough attainment in the obese population.^73^

#### 3.3.5 Patients with impaired renal function

The JPN guideline^3^ summarizes evidence and present specific recommendations for the subpopulation of patients with impaired renal function (Table 8).

**Table 8:** Guidelines review of impaired renal function patients' considerations

Question	Answer (LoE/GoR)	Guideline
Patients with impaired renal function	1. Standard or reduced single doses are given every 24 h or at even longer intervals according to renal function. (C/1)	2013, JPN^3^
	2. To facilitate rapid attainment of target trough concentration, experts recommend an initial loading dose regardless of renal function. (C/1)	
	3. As no nomogram predicts vancomycin concentrations precisely especially in patients with impaired renal function, dose should be adjusted individually based on measured vancomycin concentrations. (B/1)	

GoR, grade of recommendation; JPN‐Japanese; LoE, Level of evidence.

The Ctroughs of 62.9% patients with high creatinine clearance (Clcr) were <10 mg/L.^74^ Since augmented renal clearance was significantly associated with subtherapeutic vancomycin concentrations, it was necessary to devise adjusted dosage regimens for these patients, based on Clcr values.^74^

The “PPK Model review” in patients with impaired renal function found seven studies: two general, three on hemodialysis, one on peritoneal dialysis and one on continuous hemofiltration patient). Four of those studies were excluded as they were not population models or they were developed without using nonlinear modeling of mixed effects methods. The remaining three studies proposing PPK for patients with impaired renal are described in Table 9.

**Table 9:** Population pharmacokinetic models developed for patients with impaired renal function

Patients	N	Pharmacokinetic model	Pharmacokinetic parameters	Covariates	Reference
Impaired Renal Function	27	Two‐compartment	Vd = 0.14 L/kg; K_el_ = 0.47/h; k_12_ = 1.5/h; K_21_ = 0.53/h	ABW (covariate Vd)	75
Hemodyalisis	26	Two‐compartment	Vd = 0.105L/kg; CLDV = 0.336 × CLDBUN, Residual interdialytic clearance = 2.25 mL/min (if Clcr < 2 mL/min)	Residual interdialytic clearance: If Clcr >2 mL/min = 2.25 mL/min + 0.59 × Clcr	76
Peritoneal dyalisis	10	Two‐compartment	Cl = 0.22 L/h; V1 = 41.20 L; Cl_a_ = 0.51 L/h	None	77

ABW, actual body weight; Cl, vancomycin clearance; Cla, clearance intercompartment (peritoneal and systemic); Clcr, creatinine clearance; CLDBUN, urea filter clearance; CLDV, vancomycin filter clearance; Cr, creatinine; N, sample size; V1, volume of central compartment; Vd, volume of distribution.

- Hemodialysis patients
  The JPN guideline^3^ summarizes evidence and presents recommendations for the subpopulation of patients undergoing hemodialysis (Table 10).
  There is considerable variation in vancomycin pharmacokinetics in patients undergoing hemodialysis.^78^ Attention must be paid to the reliability of several empiric dosing recommendations derived from small pharmacokinetic studies in heterogeneous populations. Follow‐up TDM is suggested as essential to ensure that concentrations remain within the target range in these patients.^78^
  Pharmacokinetic variables of prolonged distribution phase, redistribution phase and rebound effect after completion of hemodialysis include: patient weight, residual renal function, and nonrenal clearance. Optimal vancomycin dosing recommendations are needed, but clinicians should always consider patient‐specific variables, timing of administration and of sample collection and technical aspects of the dialysis procedure. Individualized vancomycin dosing regimens and TDM are necessary for patients receiving intermittent hemodialysis to ensure that optimal serum vancomycin levels are reached to adequately treat an infection.^79^ Vancomycin removal during a typical 8‐hour sustained low‐efficiency dialysis (SLED) treatment approaches 36%.^80^ SLED patients are, therefore, at risk for undertreatment of their infections. A re‐dosing strategy should be considered (with at least 500 mg in most patients at SLED completion) if the estimated/measured predialysis level of vancomycin is 20‐30 mg/L. As such, TDM is an essential part of any dosing scheme in dialysis patients, until further studies are carried out.^80^
  Table 9 describes the PPK models developed for hemodyalisis patients found in the “PPK Model review”.
  **Table 10:** Guidelines review of patients receiving renal replacement therapy

Question	Answer (LoE/GoR)	Guideline
Patients receiving hemodialysis	1. Initial dose of 15‐25 mg/kg (as actual body weight) is recommended. As an initial dose of 15 mg/kg may not be adequate to achieve recommended trough concentrations, experts recommend that a loading dose of 20‐25 mg/kg should be administered. (C/1) 2. As a greater amount of vancomycin is removed during hemodyalisis, doses of 500 mg (7.5‐10 mg/kg) after each dialysis treatment are given as maintenance doses. (C/1) 3. Weekly vancomycin dosing results in subtherapeutic serum levels and should be abandoned in a high‐flux setting. (D/2) 4. Achievement of a steady‐state concentration is delayed. Although there is no evidence concerning the timing of TDM, the committee recommend that TDM is performed within 1 week after the start of therapy. (C/1) 5. There is no consensus concerning the necessity of follow‐up TDM in whom the dosage regimen was not altered. (Unresolved issue) 6. Blood samples for TDM should be drawn before dialysis treatment. Because of the rebound phenomenon, trough levels immediately after the completion of hemodialysis do not reflect the exact drug concentrations of patients. (C/1) 7. Although the maintenance of trough concentrations of <20 mg/L is desirable, there is no consensus concerning the concentrations causing adverse events. (Unresolved issue)	2013, JPN^3^
Patients receiving continuous renal replacement therapy	1. An initial dose of 15‐20 mg/kg (as actual body weight) is generally administered. Some experts recommend higher loading dose is required to achieve target trough concentrations. (C/1) 2. As a great amount of vancomycin is removed during continuous venovenous hemodiafiltration doses of 500 mg (7.5‐10 mg/kg) are given every 24 h as maintenance doses. It is recommended to adjust the doses according to the result of TDM. (C/1) 3. In patients with residual renal function in whom the main purpose of this therapy is removal of several mediators that cause detrimental effects during sepsis, increased vancomycin dosing may be required according to the results of TDM. (C/1)	2013, JPN^3^
Patients receiving continuous ambulatory peritoneal dialysis	1. Intraperitoneal vancomycin is well absorbed and therapeutic concentration in serum can be achieved over 1 week with single intraperitoneal administration (ie 30 mg/kg). (B/1)	2013, JPN^3^
	2. To treat peritonitis related to this treatment, doses of 15‐30 g/kg are given intraperitoneally every 5‐7 days in anuric patients. For patients with residual renal function, the doses are increased by 25%. (B/1)	

GoR, grade of recommendation; JPN, Japanese; LoE, level of evidence.
- Patients receiving continuous renal replacement therapy
  The JPN guideline^3^ summarizes evidence and presents recommendations for the subpopulation of patients receiving continuous renal replacement therapy (Table 10).
  Extracorporeal clearance of drugs increased with higher‐intensity continuous renal replacement therapy. This increase was significant for vancomycin. In these patients, there is great variability in antibiotic pharmacokinetics, which complicates an empirical approach to dosing and suggests the need for TDM.^81^
  CI produced more frequently therapeutic vancomycin levels and less frequently subtherapeutic levels compared to intermittent infusion. However, therapeutic levels were achieved infrequently by either dosing method. Given equivalent TDM costs and the lack of a clear clinical benefit, the role of CI remains to be defined, in spite of practical and theoretical advantages, particularly in burn patients.^82^
- Patients receiving ambulatory peritoneal dialysis
  The JPN guideline^3^ summarizes evidence and presents recommendations for this subpopulation (Table 10).
  Clinical outcomes of gram‐positive and culture‐negative peritonitis were not associated with either the frequency or levels of serum vancomycin measurements in the first week of treatment when vancomycin is dosed according to International Society for Peritoneal Dialysis.^83^
  Table 9 describes the PPK models developed for this subpopulation.

#### 3.3.6 Burn patients

None of the evaluated international guidelines presents defined recommendations for this subpopulation. The literature review identified a study that shows that higher clearance and lower serum vancomycin concentrations in patients with severe burns may increase the risk of suboptimal bactericidal action and development of resistance, highlighting the need for dose individualization.^84^

The “PPK Model review” found only one study in this subpopulation which is described in Table 11.

**Table 11:** Population pharmacokinetic models developed for burn patients

N	Pharmacokinetic model	Pharmacokinetic parameters	Covariates	Reference
37	Two‐compartment	Cl = 4.7L/h; V1 = 68.4 L; V2 = 73 L; Q = 4.54 L/h	CL = 4.7 × (Clcr/6.53); V1 = 68.4 × (WT/70) − 33.1 × BURN; V2 = 73 × (WT/70)	84

Cl, clearance; Clcr, creatinine clearance; N, sample size; Q, Intercompartmental clearance; V1, volume of central compartment; V2, volume of peripheric compartment; WT, weight.

#### 3.3.7 Hematologic patients

None of the evaluated international guidelines presents defined recommendations for this subpopulation. The literature review showed that this is a not well explored issue. However, it is recognized that most patients with neutropenia have augmented Clvan. A small group of patients that received vancomycin during two episodes, showed reversible augmented Clvan in the nonneutropenic period. This indicates the importance of increasing the vancomycin daily dose in 30% in patients with neutropenia (15 mg/kg, 2*x*/*d* to 13 mg/kg, 3*x*/*d*).

Frequent TDM in patients with neutropenia can help prevent therapy failure due to low AUCs and toxicity due to high vancomycin Ctroughs.^85^ Recently, Suzuki and co‐workers had proposed a target Ctrough of 11.5 mg/mL for febrile neutropenia in patients with hematological malignancies.^86^ The high‐dose, once‐daily vancomycin nomogram attained trough levels greater than 10 mg/L in only 21% of patients with leukemia and a substantial number of adverse drug reactions were observed leading to the nonrecommendation of such regimen for outpatient therapy.^87^

The “PPK Model review” found three studies in hematological patients. One was excluded because it was not a population model and the remaining two are described in Table 12.

**Table 12:** Population pharmacokinetic models developed for hematologic patients

N	Pharmacokinetic model	Pharmacokinetic parameters	Covariates	Reference
25	Two‐compartment	Vc = 15 L/65 kg; Vdss = 38.9 L/65 kg; Cl_distribution_ = 9.32 L/h/65 kg	Clcr (covariate of Cl)	88
70 Children	One‐compartment	Cl = 4.37 L/h; Vd = 119 L	Cl = 4.37 × (WT/20.2)^0.677^ × (Clcr/191)^1.03^ Vd = 119 × (WT/20.2)^0.838^	89

Cl, clearance; Clcr, creatinine clearance; N, sample size; Q, intercompartmental clearance; Vc, volume of central compartment; Vp, volume of peripheric compartment; WT, weight.

- Transplanted patients
  Current vancomycin dose regimens do not lead to recommended therapeutic serum concentrations in patients undergoing hematopoietic stem cell transplantation. Large variation in vancomycin pharmacokinetic parameters was observed among these patients, which further strenghthen the need for TDM and individualization of vancomycin dosing in this subpopulation.^90^

#### 3.3.8 Neurosurgery patients

Adult neurosurgical ICU patients showed a significantly elevated Clvan (0.104  ±  0.036  L/h/kg).^92^ Augmented Clvan should be considered when determining vancomycin doses in neurosurgical patients.^91^ Two dosing equations were derived to achieve optimal serum vancomycin concentrations for this subpopulation.^92^ Further research using TDM in the management of CNS infections, in this setting, in addition to work defining plasma and cerebrospinal fluid (CSF) concentrations associated with antibacterial efficacy and toxicity is mandatory.^93^

The “PPK Model review” in neurosurgical patients found only one study that is described in Table 13.

**Table 13:** Population pharmacokinetic models developed for neurosurgery patients

N	Pharmacokinetic model	Pharmacokinetic parameters	Covariates	Reference
25	Three‐compartment	V1 = 15.16 L; V2 = 46.10 L; V_CSF_ = 0.14 L; Q = 3.97 L/h; Q_CSF_ = 0.006 L/h; Cl = 7.98 L/h; Cl_CSF_ = 0.038 L/h	CSF albumin level	94

Cl CSF, cerebrospinal clearance; Cl, clearance; N, sample size; Q, intercompartmental distribution; QCSF, cerebrospinal distribution; V1, volume of central compartment; V2, volume of peripheric compartment; VCSF, volume of cerebrospinal fluid.

- with spinal medulla lesions
  Vancomycin dose selection in patients with spinal cord injury (SCI) is challenging due to difficulties in accurately estimating renal function in these patients.^95^ A recent study suggests that the use of the Chronic Kidney Disease Epidemiology Collaboration cystatin C equation may improve initial vancomycin dosing in the SCI population.^95^
- with meningitis
  Vancomycin penetrates the blood‐brain barrier poorly. Therefore, determination of vancomycin in CSF is rarely performed. Limited data are available on intraventricular (IVT) vancomycin dosing for meningitis. CSF output and time from dose correlated with CSF concentrations and no relationship concerning CSF protein, white blood cell count or glucose was found.^96^ Optimal regimens in this subpopulation are still unclear, and dosing of IVT vancomycin requires intricate consideration of patient specific factors and their impact on CNS pathophysiology. Higher quality clinical trials are necessary to characterize the disposition of vancomycin within the CNS, and to develop models for various pathophysiological conditions to facilitate understanding alterations of pharmacokinetic and pharmacodynamic parameters.^97^

#### 3.3.9 Other populations

- Severe acute pancreatitis
  Vancomycin Ctroughs were significantly reduced in this subpopulation and, therefore, patients with severe acute pancreatitis need higher doses to ensure clinical effects.^98^
- Trauma patients
  Vancomycin pharmacokinetics in this subpopulation is best described by a two‐compartment open model; Clcr was related to Clvan (0.49 L/h) and decreases in the presence of furosemide (0.34 L/h). ABW influenced both the central (V1 = 0.74 L/kg) and peripheral Vd (V2 = 5.9 L/kg), but patients with age >65 years showed a larger V1 (1.07 L/kg).^99^
- Extracorporeal membrane oxygenation (ECMO)
  ECMO alters vancomycin pharmacokinetics in neonates. Data in adults is limited. Clvan in patients receiving ECMO with a roller pump was significantly lower than that in the matched cohort.^100^ As a result of drug sequestration and increased Vd, the ECMO procedure might lead to a decrease in drug concentrations. Vancomycin concentration remained unchanged in the ex‐vivo ECMO circuit primed with whole human blood.^101^
  The literature review of PPK models found only one study, described in Table 14.
  **Table 14:** Population pharmacokinetic models developed for ECMO patients

N	Pharmacokinetic model	Pharmacokinetic parameters	Covariates	Reference
11	Two‐ compartment	Cl = 3.7 L/h; V1 = 31.8 L; V2 = 57.1 L	Cl = 3.7 × Cl_CRRT_ × Cl_NoCRRT_	102

Cl, clearance of vancomycin; CRRT, continuous renal replacement therapy; ECMO, extracorporeal membrane oxygenation; N, sample size; V1, volume of central compartment; V2, volume of peripheric compartment.
- Vascular surgery
  The target concentration (10‐25 mg/L) was achieved in 81% of all samples collected in one study of vascular surgery patients.^103^ All patients achieved target concentrations at one or more‐time points.
  The regimen employed provided appropriate concentrations at the time of intervention. No potentially toxic concentrations or adverse reactions to vancomycin were reported in patients undergoing vascular surgery. Vancomycin given as CI delivers adequate serum concentrations.^103^

## 4 FUTURE PERSPECTIVES

Despite the availability of consensus guideline recommendations, practices for dosing and monitoring of vancomycin are not universally applied.^104^

This review has gathered additional evidence that TDM has clinical relevance in several patient subpopulations (neonates, pediatric and with renal impairment) but there is still lack of research concerning other subpopulations (neurosurgical, oncological, cystic fibrosis).

An updated review of PPK models for specific subpopulations was carried out and models have been summarized for future reference/research and TDM refinements. Currently, most of these models have not been prospectively validated and TDM methodologies adaptations for specific populations are still not consensual. The use of dose adjustment methodologies based on PPK models and Bayesian estimation of parameters seems to gather the higher scientific consensus.

In the future, well designed prospective studies should be carried out to demonstrate the relevance of TDM, validate PPK models in clinical settings and find consensual refinement adaptations of TDM methodologies for specific patient subpopulations using vancomycin.

## 5 COMPLIANCE WITH ETHICAL STANDARDS

The authors certify that they have no affiliations with or involvement in any organization or entity with any financial interest in the subject matter or materials discussed in this manuscript.

## CONFLICT OF INTEREST

The authors hereby confirm that they do not have any conflicts of interest to declare.

## References

[1] Ye ZK , Li C , Zhai SD . Guidelines for therapeutic drug monitoring of vancomycin: a systematic review. PLoS ONE. 2014;9:e99044. 24932495 10.1371/journal.pone.0099044 PMC4059638

[2] Rybak M , Lomaestro B , Rotschafer JC , et al. Therapeutic monitoring of vancomycin in adult patients: a consensus review of the American Society of Health‐System Pharmacists, the Infectious Diseases Society of America, and the Society of Infectious Diseases Pharmacists. AJHP. 2009;66:82‐98. 19106348 10.2146/ajhp080434

[3] Matsumoto K , Takesue Y , Ohmagari N , et al. Practice guidelines for therapeutic drug monitoring of vancomycin: a consensus review of the Japanese Society of Chemotherapy and the Japanese Society of Therapeutic Drug Monitoring. J. Infect. Chemother. 2013;19:365‐380. 23673472 10.1007/s10156-013-0599-4

[4] Ye ZK , Chen YL , Chen K , et al. Therapeutic drug monitoring of vancomycin: a guideline of the Division of Therapeutic Drug Monitoring, Chinese Pharmacological Society. J. Antimicrob. Chemother. 2016;71:3020‐3025. 27494905 10.1093/jac/dkw254

[5] Guyatt GH , Oxman AD , Vist GE , et al. GRADE: an emerging consensus on rating quality of evidence and strength of recommendations. BMJ. 2008;336:924‐926. 18436948 10.1136/bmj.39489.470347.AD PMC2335261

[6] Ye ZK , Tang HL , Zhai SD . Benefits of therapeutic drug monitoring of vancomycin: a systematic review and meta‐analysis. PLoS ONE. 2013;8:e77169. 24204764 10.1371/journal.pone.0077169 PMC3799644

[7] Cardile AP , Tan C , Lustik MB , et al. Optimization of time to initial vancomycin target trough improves clinical outcomes. SpringerPlus. 2015;4:364. 26203410 10.1186/s40064-015-1146-9 PMC4506278

[8] Charmillon A , Novy E , Agrinier N , et al. The ANTIBIOPERF study: a nationwide cross‐sectional survey about practices for beta‐lactam administration and therapeutic drug monitoring among critically ill patients in France. Clin. Microbiol. Infect. 2016;22:625‐631. 27145210 10.1016/j.cmi.2016.04.019

[9] Roustit M , Francois P , Sellier E , et al. Evaluation of glycopeptide prescription and therapeutic drug monitoring at a university hospital. Scand. J. Infect. Dis. 2010;42:177‐184. 20001224 10.3109/00365540903413614

[10] Swartling M , Gupta R , Dudas V , Guglielmo BJ . Short term impact of guidelines on vancomycin dosing and therapeutic drug monitoring. Int. J. Clin. Pharm. 2012;34:282‐285. 22331444 10.1007/s11096-012-9614-6

[11] Crowley RK , Fitzpatrick F , Solanki D , FitzGerald S , Humphreys H , Smyth EG . Vancomycin administration: the impact of multidisciplinary interventions. J. Clin. Pathol. 2007;60:1155‐1159. 17293388 10.1136/jcp.2006.044727 PMC2014864

[12] Thalakada R , Legal M , Lau TT , Luey T , Batterink J , Ensom MH . Development and validation of a novel vancomycin dosing nomogram for achieving high‐target trough levels at 2 canadian teaching hospitals. Can. J. Hosp. Pharm. 2012;65:180‐187. 22783028 10.4212/cjhp.v65i3.1140 PMC3379824

[13] Nakayama H , Echizen H , Tanaka M , Sato M , Orii T . Reduced vancomycin clearance despite unchanged creatinine clearance in patients treated with vancomycin for longer than 4 weeks. Ther. Drug Monit. 2008;30:103‐107. 18223471 10.1097/FTD.0b013e318164f781

[14] Avent ML , Vaska VL , Rogers BA , et al. Vancomycin therapeutics and monitoring: a contemporary approach. Intern. Med. J. 2013;43:110‐119. 23185970 10.1111/imj.12036

[15] Hiraki Y , Onga T , Mizoguchi A , Tsuji Y . Investigation of the prediction accuracy of vancomycin concentrations determined by patient‐specific parameters as estimated by Bayesian analysis. J. Clin. Pharm. Ther. 2010;35:527‐532. 20831677 10.1111/j.1365-2710.2009.01126.x

[16] Deng C , Liu T , Zhou T , et al. Initial dosage regimens of vancomycin for Chinese adult patients based on population pharmacokinetic analysis. Int. J. Clin. Pharmacol. Ther. 2013;51:407‐415. 23458230 10.5414/CP201842

[17] Yamamoto M , Kuzuya T , Baba H , Yamada K , Nabeshima T . Population pharmacokinetic analysis of vancomycin in patients with gram‐positive infections and the influence of infectious disease type. J. Clin. Pharm. Ther. 2009;34:473‐483. 19583681 10.1111/j.1365-2710.2008.01016.x

[18] Thomson AH , Staatz CE , Tobin CM , Gall M , Lovering AM . Development and evaluation of vancomycin dosage guidelines designed to achieve new target concentrations. J. Antimicrob. Chemother. 2009;63:1050‐1057. 19299472 10.1093/jac/dkp085

[19] Bakke V , Sporsem H , Von der Lippe E , et al. Vancomycin levels are frequently subtherapeutic in critically ill patients: a prospective observational study. Acta Anaesthesiol. Scand. 2017;61:627‐635. 28444760 10.1111/aas.12897 PMC5485054

[20] Tabah A , De Waele J , Lipman J , et al. The ADMIN‐ICU survey: a survey on antimicrobial dosing and monitoring in ICUs. J. Antimicrob. Chemother. 2015;70:2671‐2677. 26169558 10.1093/jac/dkv165

[21] Jeurissen A , Sluyts I , Rutsaert R . A higher dose of vancomycin in continuous infusion is needed in critically ill patients. Int. J. Antimicrob. Agents. 2011;37:75‐77. 21074374 10.1016/j.ijantimicag.2010.09.004

[22] Roberts JA , Lipman J , Blot S , Rello J . Better outcomes through continuous infusion of time‐dependent antibiotics to critically ill patients? Curr. Opin. Crit. Care. 2008;14:390‐396. 18614901 10.1097/MCC.0b013e3283021b3a

[23] Hirai K , Ishii H , Shimoshikiryo T , et al. Augmented renal clearance in patients with febrile neutropenia is associated with increased risk for subtherapeutic concentrations of vancomycin. Ther. Drug Monit. 2016;38:706‐710. 27681114 10.1097/FTD.0000000000000346

[24] Oami T , Hattori N , Matsumura Y , et al. The effects of fasting and massive diarrhea on absorption of enteral vancomycin in critically ill patients: a retrospective observational study. Front. Med. 2017;4:70. 10.3389/fmed.2017.00070 PMC5462912 28642864

[25] Hahn A , Frenck RW Jr , Allen‐Staat M , Zou Y , Vinks AA . Evaluation of target attainment of vancomycin area under the curve in children with methicillin‐resistant *Staphylococcus aureus* bacteremia. Ther. Drug Monit. 2015;37:619‐625. 26378371 10.1097/FTD.0000000000000190 PMC4576725

[26] Buyle FM , Decruyenaere J , De Waele J , et al. A survey of beta‐lactam antibiotics and vancomycin dosing strategies in intensive care units and general wards in Belgian hospitals. Eur. J. Clin. Microbiol. Infect. Dis. 2013;32:763‐768. 23271675 10.1007/s10096-012-1803-7

[27] Tafelski S , Nachtigall I , Troeger U , et al. Observational clinical study on the effects of different dosing regimens on vancomycin target levels in critically ill patients: continuous versus intermittent application. J. Infect. Public Health. 2015;8:355‐363. 25794497 10.1016/j.jiph.2015.01.011

[28] Saugel B , Gramm C , Wagner JY , et al. Evaluation of a dosing regimen for continuous vancomycin infusion in critically ill patients: an observational study in intensive care unit patients. J. Crit. Care. 2014;29:351‐355. 24456810 10.1016/j.jcrc.2013.12.007

[29] Eldemiry EM , Sabry NA , Abbassi MM , Abdel Shafy SS , Mokhtar MS , Abdel Bary A . A specially tailored vancomycin continuous infusion regimen for renally impaired critically ill patients. SAGE Open Med. 2013;1:2050312113507921. 26770686 10.1177/2050312113507921 PMC4687768

[30] Chuma M , Makishima M , Imai T , et al. Duration of systemic inflammatory response syndrome influences serum vancomycin concentration in patients with sepsis. Clin. Ther. 2016;38:2598‐2609. 27836495 10.1016/j.clinthera.2016.10.009

[31] Roberts JA , Taccone FS , Udy AA , Vincent JL , Jacobs F , Lipman J . Vancomycin dosing in critically ill patients: robust methods for improved continuous‐infusion regimens. Antimicrob. Agents Chemother. 2011;55:2704‐2709. 21402850 10.1128/AAC.01708-10 PMC3101407

[32] de Gatta MD , Revilla N , Calvo MV , Domínguez‐Gil A , Navarro AS . Pharmacokinetic/pharmacodynamic analysis of vancomycin in ICU patients. Intensive Care Med. 2007;33:279‐285. 17165021 10.1007/s00134-006-0470-5

[33] Arfa P , Karimi A , Rafiei Tabatabaei S , Fahimzad A , Armin S , Sistanizad M . A prospective study to assess vancomycin serum concentrations inpediatric patients with current dosing guidelines. IJPR. 2016;15:341‐346. 27610175 PMC4986101

[34] Hirai K , Ihara S , Kinae A , et al. Augmented renal clearance in pediatric patients with febrile neutropenia associated with vancomycin clearance. Ther. Drug Monit. 2016;38:393‐397. 27172381 10.1097/FTD.0000000000000270

[35] Kishk OA , Lardieri AB , Heil EL , Morgan JA . Vancomycin AUC/MIC and corresponding troughs in a pediatric population. JPPT. 2017;22:41‐47. 28337080 10.5863/1551-6776-22.1.41 PMC5341531

[36] Balch AH , Constance JE , Thorell EA , et al. Pediatric vancomycin dosing: trends over time and the impact of therapeutic drug monitoring. J. Clin. Pharmacol. 2015;55:212‐220. 25264036 10.1002/jcph.402 PMC5641452

[37] Moffett BS , Edwards MS . Analysis of vancomycin therapeutic drug monitoring trends at pediatric hospitals. Pediatr. Infect. Dis. J. 2013;32:32‐35. 22926218 10.1097/INF.0b013e31826fd98d

[38] Heble DE Jr , McPherson C , Nelson MP , Hunstad DA . Vancomycin trough concentrations in overweight or obese pediatric patients. Pharmacotherapy. 2013;33:1273‐1277. 23798327 10.1002/phar.1321

[39] Guilhaumou R , Marsot A , Dupouey J , et al. Pediatric patients with solid or hematological tumor disease: vancomycin population pharmacokinetics and dosage optimization. Ther. Drug Monit. 2016;38:559‐566. 27631462 10.1097/FTD.0000000000000318

[40] Le J , Ngu B , Bradley JS , et al. Vancomycin monitoring in children using bayesian estimation. Ther. Drug Monit. 2014;36:510‐518. 24452067 10.1097/FTD.0000000000000039 PMC4101060

[41] Hahn A , Frenck RW Jr , Zou Y , Vinks AA . Validation of a pediatric population pharmacokinetic model for vancomycin. Ther. Drug Monit. 2015;37:413‐416. 25423413 10.1097/FTD.0000000000000153 PMC4431905

[42] Wrishko RE , Levine M , Khoo D , Abbott P , Hamilton D . Vancomycin pharmacokinetics and Bayesian estimation in pediatric patients. Ther. Drug Monit. 2000;22:522‐531. 11034256 10.1097/00007691-200010000-00004

[43] Lamarre P , Lebel D , Ducharme MP . A population pharmacokinetic model for vancomycin in pediatric patients and its predictive value in a naive population. Antimicrob. Agents Chemother. 2000;44:278‐282. 10639350 10.1128/aac.44.2.278-282.2000 PMC89671

[44] Stockmann C , Sherwin CM , Zobell JT , et al. Population pharmacokinetics of intermittent vancomycin in children with cystic fibrosis. Pharmacotherapy. 2013;33:1288‐1296. 23824677 10.1002/phar.1320

[45] Bhongsatiern J , Stockmann C , Roberts JK , et al. Evaluation of vancomycin use in late‐onset neonatal sepsis using the area under the concentration‐time curve to the minimum inhibitory concentration >/=400 target. Ther. Drug Monit. 2015;37:756‐765. 26562817 10.1097/FTD.0000000000000216 PMC5641451

[46] Frymoyer A , Hersh AL , El‐Komy MH , et al. Association between vancomycin trough concentration and area under the concentration‐time curve in neonates. Antimicrob. Agents Chemother. 2014;58:6454‐6461. 25136027 10.1128/AAC.03620-14 PMC4249374

[47] Marques‐Minana MR , Saadeddin A , Peris JE . Population pharmacokinetic analysis of vancomycin in neonates. A new proposal of initial dosage guideline. Br. J. Clin. Pharmacol. 2010;70:713‐720. 21039765 10.1111/j.1365-2125.2010.03736.x PMC2997311

[48] Capparelli EV , Lane JR , Romanowski GL , et al. The influences of renal function and maturation on vancomycin elimination in newborns and infants. J. Clin. Pharmacol. 2001;41:927‐934. 11549096 10.1177/00912700122010898

[49] Rodvold KA , Gentry CA , Plank GS , Kraus DM , Nickel E , Gross JR . Bayesian forecasting of serum vancomycin concentrations in neonates and infants. Ther. Drug Monit. 1995;17:239‐246. 7624919 10.1097/00007691-199506000-00005

[50] Mehrotra N , Tang L , Phelps SJ , Meibohm B . Evaluation of vancomycin dosing regimens in preterm and term neonates using Monte Carlo simulations. Pharmacotherapy. 2012;32:408‐419. 22488303 10.1002/j.1875-9114.2012.01029.x

[51] McDade EJ , Hewlett JL , Moonnumakal SP , Baker CJ . Evaluation of vancomycin dosing in pediatric cystic fibrosis patients. JPPT. 2016;21:155‐161. 27199623 10.5863/1551-6776-21.2.155 PMC4869773

[52] Jacqz‐Aigrain E , Leroux S , Zhao W , van den Anker JN , Sharland M . How to use vancomycin optimally in neonates: remaining questions. Expert Rev. Clin. Pharmacol. 2015;8:635‐648. 26289222 10.1586/17512433.2015.1060124

[53] Roberts JK , Stockmann C , Constance JE , et al. Pharmacokinetics and pharmacodynamics of antibacterials, antifungals, and antivirals used most frequently in neonates and infants. Clin. Pharmacokinet. 2014;53:581‐610. 24871768 10.1007/s40262-014-0147-0

[54] Hammer BM , Lardieri AB , Morgan JA . Appropriate use of vancomycin in NICU despite free‐for‐all policy. JPPT. 2016;21:207‐212. 27453698 10.5863/1551-6776-21.3.207 PMC4956328

[55] Pauwels S , Allegaert K . Therapeutic drug monitoring in neonates. Arch. Dis. Child. 2016;101:377‐381. 26803050 10.1136/archdischild-2013-305309

[56] Padari H , Oselin K , Tasa T , Metsvaht T , Loivukene K , Lutsar I . Coagulase negative staphylococcal sepsis in neonates: do we need to adapt vancomycin dose or target? BMC Pediatr. 2016;16:206. 27931193 10.1186/s12887-016-0753-0 PMC5146818

[57] Vandendriessche A , Allegaert K , Cossey V , Naulaers G , Saegeman V , Smits A . Prospective validation of neonatal vancomycin dosing regimens is urgently needed. Curr. Ther. Res. Clin. Exp. 2014;76:51‐57. 25061483 10.1016/j.curtheres.2014.06.001 PMC4099512

[58] Pacifici GM , Allegaert K . Clinical pharmacokinetics of vancomycin in the neonate: a review. Clinics. 2012;67:831‐837. 22892931 10.6061/clinics/2012(07)21 PMC3400177

[59] Badran EF , Shamayleh A , Irshaid YM . Pharmacokinetics of vancomycin in neonates admitted to the neonatology unit at the Jordan University Hospital. Int. J. Clin. Pharmacol. Ther. 2011;49:252‐257. 21429439 10.5414/CP201456

[60] Oudin C , Vialet R , Boulamery A , Martin C , Simon N . Vancomycin prescription in neonates and young infants: toward a simplified dosage. Arch. Dis. Child. Fetal Neonatal Ed. 2011;96:F365‐F370. 21378399 10.1136/adc.2010.196402

[61] Gwee A , Cranswick N , Metz D , et al. Neonatal vancomycin continuous infusion: still a confusion? Pediatr. Infect. Dis. J. 2014;33:600‐605. 24378952 10.1097/INF.0000000000000243

[62] Zhao W , Lopez E , Biran V , Durrmeyer X , Fakhoury M , Jacqz‐Aigrain E . Vancomycin continuous infusion in neonates: dosing optimisation and therapeutic drug monitoring. Arch. Dis. Child. 2013;98:449‐453. 23254142 10.1136/archdischild-2012-302765

[63] Jacqz‐Aigrain E , Zhao W , Sharland M , van den Anker JN . Use of antibacterial agents in the neonate: 50 years of experience with vancomycin administration. Semin. Fetal Neonatal. Med. 2013;18:28‐34. 23137927 10.1016/j.siny.2012.10.003

[64] Samardzic J , Allegaert K , Wilbaux M , Pfister M , van den Anker JN . Quantitative clinical pharmacology practice for optimal use of antibiotics during the neonatal period. Expert Opin. Drug Metab. Toxicol. 2016;12:367‐375. 26817821 10.1517/17425255.2016.1147559

[65] Marsot A , Vialet R , Boulamery A , Bruguerolle B , Simon N . Vancomycin: predictive performance of a population pharmacokinetic model and optimal dose in neonates and young infants. Clin. Pharmacol. Drug Develop. 2012;1:144‐151. 10.1177/2160763X12456843 27121456

[66] Leroux S , Jacqz‐Aigrain E , Biran V , et al. Clinical utility and safety of a model‐based patient‐tailored dose of vancomycin in neonates. Antimicrob. Agents Chemother. 2016;60:2039‐2042. 26787690 10.1128/AAC.02214-15 PMC4808207

[67] Bel Kamel A , Bourguignon L , Marcos M , Ducher M , Goutelle S . Is trough concentration of vancomycin predictive of the area under the curve? A clinical study in elderly patients. Ther. Drug Monit. 2017;39:83‐87. 27861313 10.1097/FTD.0000000000000359

[68] Fukumori S , Tsuji Y , Mizoguchi A , et al. Association of the clinical efficacy of vancomycin with the novel pharmacokinetic parameter area under the trough level (AUTL) in elderly patients with hospital‐acquired pneumonia. J. Clin. Pharm. Ther. 2016;41:399‐402. 27144370 10.1111/jcpt.12399

[69] Kosmisky DE , Griffiths CL , Templin MA , Norton J , Martin KE . Evaluation of a new vancomycin dosing protocol in morbidly obese patients. Hospital Pharm. 2015;50:789‐797. 10.1310/hpj5009-789 PMC4750829 26912920

[70] Tafelski S , Yi H , Ismaeel F , Krannich A , Spies C , Nachtigall I . Obesity in critically ill patients is associated with increased need of mechanical ventilation but not with mortality. J. Infect. Public Health. 2016;9:577‐585. 26754202 10.1016/j.jiph.2015.12.003

[71] Abuhasna S , Al Jundi AH . Therapeutic drug monitoring of vancomycin in an obese patient with renal insufficiency. J. Anaesthesiol. Clin. Pharmacol. 2011;27:531‐533. 22096290 10.4103/0970-9185.86601 PMC3214562

[72] Grupper M , Nicolau DP . Obesity and skin and soft tissue infections: how to optimize antimicrobial usage for prevention and treatment? Curr. Opin. Infect. Dis. 2017;30:180‐191. 28118218 10.1097/QCO.0000000000000356

[73] Hong J , Krop LC , Johns T , Pai MP . Individualized vancomycin dosing in obese patients: a two‐sample measurement approach improves target attainment. Pharmacotherapy. 2015;35:455‐463. 26011138 10.1002/phar.1588

[74] Chu Y , Luo Y , Qu L , Zhao C , Jiang M . Application of vancomycin in patients with varying renal function, especially those with augmented renal clearance. Pharm. Biol. 2016;54:2802‐2806. 27251880 10.1080/13880209.2016.1183684

[75] Hurst AK , Yoshinaga MA , Mitani GH , Foo KA , Jelliffe RW , Harrison EC . Application of a Bayesian method to monitor and adjust vancomycin dosage regimens. Antimicrob. Agents Chemother. 1990;34:1165‐1171. 2393277 10.1128/aac.34.6.1165 PMC171778

[76] Schaedeli F , Uehlinger DE . Urea kinetics and dialysis treatment time predict vancomycin elimination during high‐flux hemodialysis. Clin. Pharmacol. Ther. 1998;63:26‐38. 9465839 10.1016/S0009-9236(98)90118-7

[77] Montanes Pauls B , Alminana MA , Casabo Alos VG . Vancomycin pharmacokinetics during continuous ambulatory peritoneal dialysis in patients with peritonitis. Eur. J. Pharm. Sci. 2011;43:212‐216. 21540106 10.1016/j.ejps.2011.04.006

[78] van de Vijsel LM , Walker SA , Walker SE , Yamashita S , Simor A , Hladunewich M . Initial vancomycin dosing recommendations for critically ill patients undergoing continuous venovenous hemodialysis. Can. J. Hosp. Pharm. 2010;63:196‐206. 22478979 10.4212/cjhp.v63i3.915 PMC2901779

[79] Crew P , Heintz SJ , Heintz BH . Vancomycin dosing and monitoring for patients with end‐stage renal disease receiving intermittent hemodialysis. AJHP. 2015;72:1856‐1864. 26490819 10.2146/ajhp150051

[80] Golestaneh L , Gofran A , Mokrzycki MH , Chen JL . Removal of vancomycin in sustained low‐efficiency dialysis (SLED): a need for better surveillance and dosing. Clin. Nephrol. 2009;72:286‐291. 19825334 10.5414/cnp72286

[81] Roberts DM , Liu X , Roberts JA , et al. A multicenter study on the effect of continuous hemodiafiltration intensity on antibiotic pharmacokinetics. Crit. Care. 2015;19:84. 25881576 10.1186/s13054-015-0818-8 PMC4404619

[82] Akers KS , Cota JM , Chung KK , Renz EM , Mende K , Murray CK . Serum vancomycin levels resulting from continuous or intermittent infusion in critically ill burn patients with or without continuous renal replacement therapy. J. Burn Care Res. 2012;33:e254‐e262. 22878490 10.1097/BCR.0b013e31825042fa

[83] Stevenson S , Tang W , Cho Y , et al. The role of monitoring vancomycin levels in patients with peritoneal dialysis‐associated peritonitis. Perit. Dial. Int. 2015;35:222‐228. 24584597 10.3747/pdi.2013.00156 PMC4406318

[84] Dolton M , Xu H , Cheong E , et al. Vancomycin pharmacokinetics in patients with severe burn injuries. Burns. 2010;36:469‐476. 19875238 10.1016/j.burns.2009.08.010

[85] Omote S , Yano Y , Hashida T , et al. A retrospective analysis of vancomycin pharmacokinetics in Japanese cancer and non‐cancer patients based on routine trough monitoring data. Biol. Pharm. Bull. 2009;32:99‐104. 19122288 10.1248/bpb.32.99

[86] Suzuki Y , Tokimatsu I , Morinaga Y , et al. A retrospective analysis to estimate target trough concentration of vancomycin for febrile neutropenia in patients with hematological malignancy. Clin. Chim. Acta. 2015;440:183‐187. 25476135 10.1016/j.cca.2014.11.027

[87] Luo C , Hussaini T , Lacaria K , Yeung J , Lau TT , Broady RC . Evaluation of a once‐daily vancomycin regimen in an outpatient leukemia/bone marrow transplant clinic (OD‐VANCO Study). Can. J. Hospital Pharm. 2014;67:280‐285. 10.4212/cjhp.v67i4.1372 PMC4152967 25214659

[88] Jarkowski A 3rd , Forrest A , Sweeney RP , et al. Characterization of vancomycin pharmacokinetics in the adult acute myeloid leukemia population. J. Oncol. Pharm. Pract. 2012;18:91‐96. 21521799 10.1177/1078155211402107

[89] Zhao W , Zhang D , Fakhoury M , et al. Population pharmacokinetics and dosing optimization of vancomycin in children with malignant hematological disease. Antimicrob. Agents Chemother. 2014;58:3191‐3199. 24663023 10.1128/AAC.02564-13 PMC4068451

[90] Ghehi MT , Rezaee S , Hayatshahi A , et al. Vancomycin pharmacokinetic parameters in patients undergoing hematopoietic stem cell transplantation (HSCT). Int. J. Hematol. Ther. 2013;7:1‐9. PMC3915428 24505536

[91] Kim AJ , Lee JY , Choi SA , Shin WG . Comparison of the pharmacokinetics of vancomycin in neurosurgical and non‐neurosurgical patients. Int. J. Antimicrob. Agents. 2016;48:381‐387. 27546217 10.1016/j.ijantimicag.2016.06.022

[92] Lin Wu FL , Liu SS , Yang TY , et al. A larger dose of vancomycin is required in adult neurosurgical intensive care unit patients due to augmented clearance. Ther. Drug Monit. 2015;37:609‐618. 25627406 10.1097/FTD.0000000000000187

[93] Lonsdale DO , Udy AA , Roberts JA , Lipman J . Antibacterial therapeutic drug monitoring in cerebrospinal fluid: difficulty in achieving adequate drug concentrations. J. Neurosurg. 2013;118:297‐301. 23121433 10.3171/2012.10.JNS12883

[94] Li X , Wu Y , Sun S , et al. Population pharmacokinetics of vancomycin in postoperative neurosurgical patients. J. Pharm. Sci. 2015;104:3960‐3967. 26239933 10.1002/jps.24604

[95] DeCarolis DD , Thorson JG , Marraffa RA , Clairmont MA , Kuskowski MA . Comparison of equations with estimate renal function to predict serum vancomycin concentration in patients with spinal cord injury–does the use of cystatin C improve accuracy? Ther. Drug Monit. 2014;36:632‐639. 25222855 10.1097/FTD.0000000000000065

[96] Popa D , Loewenstein L , Lam SW , Neuner EA , Ahrens CL , Bhimraj A . Therapeutic drug monitoring of cerebrospinal fluid vancomycin concentration during intraventricular administration. J. Hospital Infect. 2016;92:199‐202. 10.1016/j.jhin.2015.10.017 26654472

[97] Ng K , Mabasa VH , Chow I , Ensom MH . Systematic review of efficacy, pharmacokinetics, and administration of intraventricular vancomycin in adults. Neurocrit. Care. 2014;20:158‐171. 23090839 10.1007/s12028-012-9784-z

[98] He J , Mao EQ , Feng J , Jiang HT , Yang WH , Chen EZ . The pharmacokinetics of vancomycin in patients with severe acute pancreatitis. Eur. J. Clin. Pharmacol. 2016;72:697‐702. 26902230 10.1007/s00228-016-2018-0

[99] Medellin‐Garibay SE , Ortiz‐Martin B , Rueda‐Naharro A , Garcia B , Romano‐Moreno S , Barcia E . Pharmacokinetics of vancomycin and dosing recommendations for trauma patients. J. Antimicrob. Chemother. 2016;71:471‐479. 26568565 10.1093/jac/dkv372

[100] Wu CC , Shen LJ , Hsu LF , Ko WJ , Wu FL . Pharmacokinetics of vancomycin in adults receiving extracorporeal membrane oxygenation. JFMA. 2016;115:560‐570. 10.1016/j.jfma.2015.05.017 26123638

[101] Lemaitre F , Hasni N , Leprince P , et al. Propofol, midazolam, vancomycin and cyclosporine therapeutic drug monitoring in extracorporeal membrane oxygenation circuits primed with whole human blood. Crit. Care. 2015;19:40. 25886890 10.1186/s13054-015-0772-5 PMC4335544

[102] Donadello K , Roberts JA , Cristallini S , et al. Vancomycin population pharmacokinetics during extracorporeal membrane oxygenation therapy: a matched cohort study. Crit. Care. 2014;18:632. 25416535 10.1186/s13054-014-0632-8 PMC4256896

[103] Payne CJ , Carmichael SJ , Stearns AT , Kingsmore DB , Byrne DS , Binning AR . Vancomycin continuous infusion as prophylaxis for vascular surgery. Ther. Drug Monit. 2009;31:786‐788. 19809387 10.1097/FTD.0b013e3181bddf70

[104] Davis SL , Scheetz MH , Bosso JA , Goff DA , Rybak MJ . Adherence to the 2009 consensus guidelines for vancomycin dosing and monitoring practices: a cross‐sectional survey of U.S. hospitals. Pharmacotherapy. 2013;33:1256‐1263. 23897602 10.1002/phar.1327

